# Functional Traits of Olive Varieties and Their Relationship with the Tolerance Level towards Verticillium Wilt

**DOI:** 10.3390/plants10061079

**Published:** 2021-05-27

**Authors:** Martina Cardoni, Jesús Mercado-Blanco, Rafael Villar

**Affiliations:** 1 Departamento de Protección de Cultivos, Instituto de Agricultura Sostenible, CSIC, Campus ‘Alameda del Obispo’ s/n, Avd. Menéndez Pidal s/n, 14004 Córdoba, Spain; mcardoni@ias.csic.es; 2 Departamento de Botánica, Ecología y Fisiología Vegetal, Universidad de Córdoba, Campus Universitario de Rabanales, 14014 Córdoba, Spain; rafael.villar@uco.es

**Keywords:** biomass allocation, breeding for resistance, dry matter content, leaf area, lignin, root architecture, SRA (specific root area), SRL (specific root length), *Verticillium dahliae*

## Abstract

Verticillium wilt of olive (VWO), caused by the soil-borne pathogen *Verticillium dahliae,* is considered one of the most important diseases affecting this tree crop. One of the best VWO management measures is the use of tolerant cultivars. Remarkably, no information is available about olive functional traits and their potential relationship with tolerance to *V. dahliae*. Twenty-five selected functional traits (for leaf, stem, root and whole plant) were evaluated in six olive varieties differing in their VWO tolerance level to identify possible links between this phenotype and functional traits’ variation. High intervarietal diversity was found among cultivars and several functional traits were related with VWO tolerance. Tolerant varieties showed higher leaf area, dry matter content (leaf, stem and plant) and mass fraction for stems, but lower for leaves. Significant differences were also detected for root functional traits, tolerant cultivars displaying larger fine root diameter and lignin content but smaller specific length and area of thick and fine roots. Correlations were found among functional traits both within varieties and between levels of tolerance/susceptibility to VWO. Associations were observed between biomass allocation, dry matter content and VWO tolerance. The most relevant difference between tolerant and susceptible cultivars was related to root system architecture.

## 1. Introduction

Olive (*Olea europaea* L. subsp. *europaea* var. *europaea*) is one of the most ancient cultivated fruit trees in the Mediterranean Basin. It constitutes an agroecosystem of major relevance for southern Europe where the cultivated area covers more than 5 million ha, with Spain, Italy and Greece standing out as the countries with the largest cropping acreages [1].

The soil borne, vascular-colonizing fungus *Verticillium dahliae* Kleb., particularly the highly virulent defoliating (D) pathotype (lineage 1A), is the causal agent of Verticillium wilt of olive (VWO). The disease is considered one of the most threatening biotic constraints in many olive growing countries [2]. A mean disease incidence of 0.4% has been reported in Spain, but in some areas it can reach up to 9%, with more than 50% of orchards affected [3]. Thus, VWO has a high socio-economic significance because of the magnitude of olive cultivation acreage. In addition, several characteristics of the pathogen make the disease very difficult to control. Certainly, *V. dahliae* can survive in the soil for many years, it has a wide host range, from annual plants to woody crops [4], and available fungicides are not effective [3].

The use of tolerant/resistant olive cultivars is a promising, environmentally friendly and economically profitable control tool for managing this disease [3,5]. Indeed, varieties displaying tolerance can be used to substitute dead trees, as rootstocks, and as a source of resistance to *V. dahliae* in breeding programs [6]. Yet, no olive cultivar has so far been reported as fully resistant to VWO, and only moderate level of tolerance has been reported for some varieties, either of local (e.g., ‘Tortosa’, ‘Palomillo’ and ‘Toruno’ [7]) or major (e.g., ‘Empeltre’, ‘Frantoio’, and ‘Koroneki’ [7,8]) economic importance. Tolerant cultivars are able to restrict the colonization of plant tissues by the pathogen, thus delaying or hindering the disease progress, increasing the recovery of infected plants, and reducing the percentage of dead plants compared with susceptible cultivars [9].

The olive–*V. dahliae* interaction has been investigated from different perspectives, which has provided good knowledge on relevant aspects of this pathosystem. Thus, advances have been achieved in aspects such as: (i) the pathogen’s colonization process [10]; (ii) the triggering of host defense-related systemic responses upon pathogen infection [11]; (iii) the changes in plant root–belowground microbiome interaction related to the presence/absence of the pathogen [5]; (iv) the chemical and physical processes taking place in roots and stems to restrict the colonization by the pathogen [12]; and (v) the effect of vessel occlusion caused by the invasion [13]. Yet, no studies have been conducted on the potential relationship between the different levels of VWO tolerance of olive cultivars and their functional traits. 

‘Plant functional trait’ is a commonly used expression in plant ecology but its actual meaning varies substantially among authors [14]. It may be understood as a surrogate of a function (e.g., specific leaf area) or as this function itself (e.g., photosynthesis). It may also be regarded as a trait that strongly influences organismal performance [15] and/or individual fitness. Violle and coworkers [14] have proposed a definition of functional trait as “*a feature measurable at the level of the individual, which does not require additional information from the environment or at any other organizational level*”. Moreover, they distinguished between performance traits (directly contributing to the fitness) and functional traits (physiological and phenological traits which impact performance traits and indirectly the fitness). Functional plant traits are widely used indicators of species’ ecological functions, as they allow us to appraise the spectrum of functional strategies in plants and their relationships with the environment [16]. The functional approach is considered a powerful tool for better understanding the diversity of plant adaptations at both genus level and among species within a genus. Recently, this type of study has deepened at the intraspecific level as well [17,18]. In many studies, the intraspecific variability of functional traits was considered negligible compared with the interspecific one [19,20]. However, growing evidence indicates that intraspecific functional variability, as well as genetic diversity, can have significant effects on community dynamics and ecosystem functioning [21,22,23]. Moreover, intraspecific functional variability can also influence community assembly and stability, thereby being essential to the fundamental processes of natural selection and speciation [19].

Plant (and more specifically root) functional traits can influence soil properties (i.e., C dynamics and sequestration, nutrient availability) as well as play a significant role in reducing crop losses due to insect and pathogen attacks, influencing the abundance and diversity of soil microbial pathogens [24]. Root traits can restrict the physical contact between the pathogen and the host, create unfavorable environments for the growth of the former, or limit its ability to initiate the infection process, thereby avoiding or hampering the disease development [25]. A disease management strategy based on the effects of plant (and in particular root) traits on soil borne microbial pathogens can thus provide alternatives to both reduce the use of fungicides and improve crop quality [24]. Recent studies have found a robust causal relationship among root anatomy, morphology and physiology, which may explain traits at whole-plant level such as plant physiology, plant height and growth [26]. Even though many studies about root structure and functioning are available, important questions on issues such as the differential response to abiotic and biotic stresses, the relationship between root anatomy and root and plant physiology, or the ecological significance of variation in root morphology are yet to be answered [26,27]. Dias and coworkers [28] showed that some root architectural traits (e.g., root depth and lateral branching) can contribute to restrict disease development caused by specific soil borne pathogens. They reported that one line (Pat 81) of the wild species of melon, *Cucumis melon* L. ssp. *agrestis*, tolerant to the soil-borne fungus *Monosporascus cannonballus*, showed higher biomass, root fresh mass, root surface area, root length, increased root branching, and lower disease lesion development compared with the susceptible commercial cultivar Piñonet. They concluded that tolerance of Pat 81 to melon vine decline was due to root architecture and growth. Román-Avilés and coworkers [29] argued that quantitative information on common bean root system traits associated with Fusarium root rot tolerance would improve selection criteria that, consequently, should be a major goal in breeding programs. In their study, root system architectural changes (i.e., adventitious roots, total root dry mass, lateral roots) were reported to respond to environmental conditions and biotic stress caused by soil borne pathogens. 

The study of woody plant roots is particularly challenging because of the maturation of the finest roots leads to tissues of different structure and function. This developmental and structural differentiation may affect the susceptibility of tissues to pathogen infection and colonization, as well as the impact of such colonization on the plant [30]. Thus, understanding how root development affects specific plant–pathogen interactions would help to better comprehend both the root disease source and the basis of the host’s susceptibility and resistance. Emmett and coworkers [30] reported that root structural and chemical changes may constrain the quantity and quality of the habitat available for a given pathogen, or shift allocation of plant defenses against its infection and colonization. In addition to the differences in structure and function derived from the root system development, differences in root susceptibility to colonization may also depend on the distribution of plant defenses within the root system [30]. For example, correlation between tissue lignification and disease resistance has been proved in different studies [31,32]. Indeed, the importance of lignin in response to many pathogens and its central role in the hypersensitive response of plants to pathogens has been reported, including olive plants [33]. Deposition of lignin, lignin-like polymers, and other wall-bound phenolic materials is reported to be a response to mechanical damage or microbial infection [34]. Lignin is a complex aromatic biopolymer that strengthens and waterproofs plant secondary cell walls, increasing plant tissues mechanical stability and rigidity [35], and creating a physical barrier against pathogens. In addition, lignin deposition is suggested to decrease the diffusion to the host of enzymes and toxins released by hyphae of pathogenic fungi, and of water and nutrients from the former to the fungus, thereby starving the intruder [34]. For these reasons, a relationship between higher lignin content in olive roots and tolerance to *V. dahliae* is predictable [12].

Many functional traits do not vary independently but rather form groups of covarying traits, sometimes known as strategy spectra (or dimensions/axes of ecological/evolutionary specialization) [36]. For instance, some studies matched the specific leaf area (SLA) with the specific root length (SRL, root length/root mass) since both traits relate to resource (light vs. water and nutrient) acquisition vs. resource conservation strategies [37]. Additionally, the relative amount of biomass presents in various organs (i.e., ‘biomass allocation’) is not fixed but may vary over time, across environments and among species [38]. Olmo and coworkers [39] found that shoot and root plant biomass were related to drought response. Thus, a decrease in shoot biomass and an increase in root biomass allowed plants to minimize water loss by transpiration and increased the efficiency of the soil exploration and water acquisition, leading to a higher probability of survival [40].

No information is yet available about plant functional traits in olive and their relation with pathogen tolerance. An accurate characterization of leaves’, stems’ and roots’ functional traits of olive genotypes/varieties differing in VWO tolerance level would be interesting. Thus, the main objectives (and hypotheses to-be-tested) of this work were: (i) to explore potential differences of 25 selected functional traits (of the leaf, stem, root and whole plant) in 6 olive varieties differing in the tolerance level to VWO; the presence of high intraspecific variability in functional traits among olive varieties would be expected. (ii) To identify possible links between the level of tolerance to *V. dahliae* and functional traits and root lignin content; the tolerance level to VWO would be related to specific functional traits and to the root lignin content. (iii) To know whether a correlation among the functional traits here considered for the different organs does exist; a strong coordination among traits in leaf, stem and root would be expected.

## 2. Results

### 2.1. Variability in Functional Traits among Olive Varieties and between VWO Susceptibility/Tolerance Level

The ANOVA analysis with ‘variety’ as factor showed significant differences in leaf functional traits among varieties for all analyzed traits. Besides, their variances were explained by olive variety diversity with values higher than 70%, the ‘Leaf mass per area’ (LMA) being the largest percentage (Table 1). Considering plant traits, the ‘Plant dry mass content’ showed the higher percentage of explained variance (89.43%), followed by ‘Stem (SMF)’ and ‘Leaf (LMF) mass fraction’, while the ‘root mass fraction’ (RMF) did not show significant differences among cultivars (*p* > 0.05). Remarkably, the analyzed root traits showed a low percentage of explained variance by variety, except for ‘Fine root average diameter’ (67.6%), ‘Thick root specific area’ (51.19%) and ‘Thick root specific length’ (45.16%).

Considering ‘tolerance’ as factor in the ANOVA analysis, we found fewer significant differences comparing leaf traits between susceptible and tolerant cultivars (Table 1). Only ‘Area’, ‘Perimeter’ and ‘Dry matter content’ (LDMC) showed significant differences between the two groups of cultivars (*p* < 0.05), with a very high percentage of the explained variance (between 87 and 97%). In this case, the plants traits ‘Plant dry matter content’ (PDMC), LMF and SMF also showed significant (*p* < 0.05) differences, explaining between 57 and 93% of the variance. In contrast to leaf traits, the analyzed root traits showed a high percentage of explained variance (>67%). The fine root traits showed significant differences (*p* < 0.05) for the ‘Diameter’ (AvgDiam Fine Root), while the thick roots showed differences (*p* < 0.01) for the ‘Specific root area’ (SRA Thick Root). Finally, both fine and thick roots showed significant differences (*p* < 0.05) for ‘Specific root length’ (SRL) between tolerant and susceptible cultivars.

### 2.2. Differences in Leaf and Stem Functional Traits

**Figure 1:**
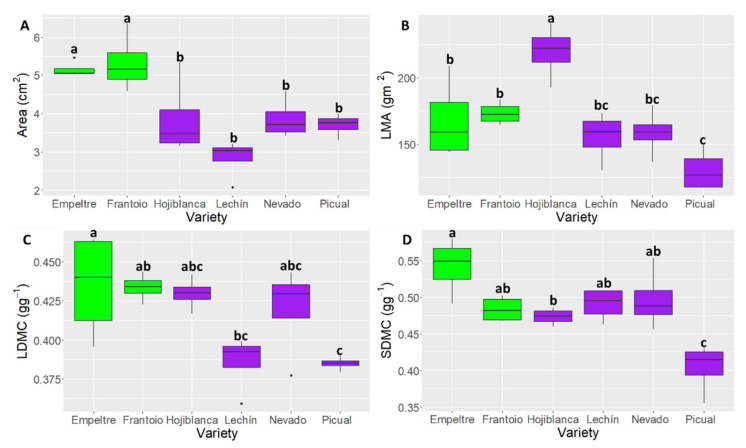
Boxplots showing median values of Area (‘leaf area’) (**A**), LMA (‘Leaf mass per area’) (**B**), LDMC (‘Leaf dry matter content’) (**C**) and SDMC (‘Stem dry matter content’) (**D**). Tolerant cultivars are represented in green color while the susceptible ones are shown in purple color. Letters indicate Tukey HSD post hoc tests at the *p* < 0.05 level, following ANOVA.

Differences among olive varieties were found for most of the leaf and stem traits (Table 1, Figure 1). Leaf ‘Area’ widely differed among varieties, with the highest values for ‘Frantoio’ (6.3 cm^2^) and the lowest values for ‘Lechín de Sevilla’ (2.1 cm^2^) (Figure 1A). Additionally, tolerant varieties showed larger leaf areas than susceptible ones (Table 1, Figure 1A. LMA varied also among varieties from 241 g·m^−2^ (‘Hojiblanca’) to 117 g·m^−2^ (‘Picual’) (Figure 1B). Overall, however, no statistical differences between tolerant and susceptible varieties were found, although ‘Empeltre’ and ‘Frantoio’ plants (tolerant) showed higher values than ‘Picual’ (in this case significant), ‘Lechín de Sevilla’ and ‘Nevado fino’ plants (all susceptible to VWO). LDMC and SDMC also differed among varieties (Figure 1C and D). Interestingly enough, tolerant cultivars showed higher (significantly in some cases) LDMC and SDMC values than susceptible cultivars. Thus, ‘Empeltre’ displayed significantly larger values of LDMC than ‘Lechín de Sevilla’ and ‘Picual’; and ‘Empeltre’ and ‘Frantoio’ showed significantly higher values of SDMC than ‘Picual’ (Figure 1C,D, Table 1). Cultivars also differed in other functional traits such as ‘Perimeter’, ‘L/W ratio’ and ‘Leaf green density’ (Table 1).

**Figure 2:**
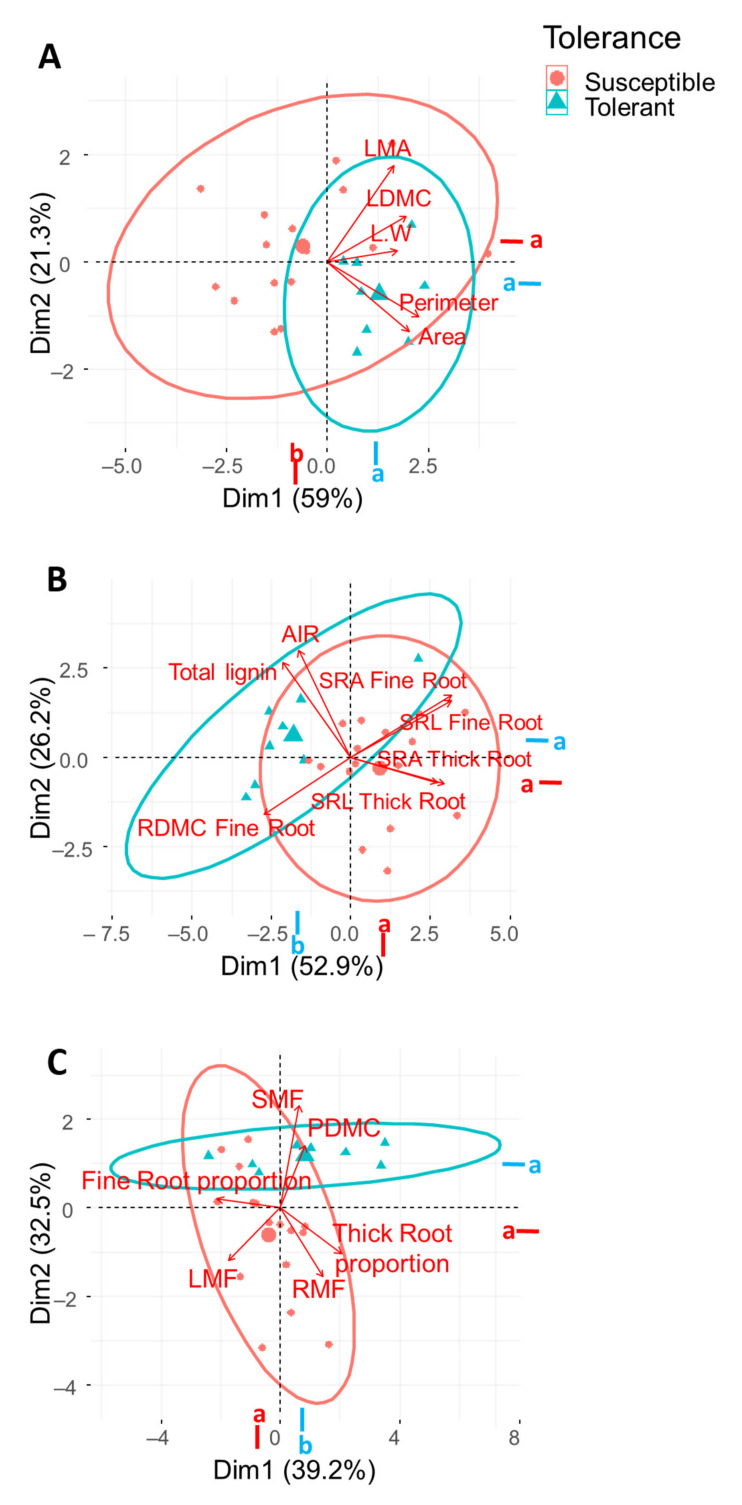
Principal Component Analysis (PCA) of leaf (**A**), root (**B**) and plant (**C**) traits performed with ‘tolerance’ as factor, and the contribution of variables on the main two axes of PCA. Different letters (a or b) indicate significant differences between groups (Tukey test, *p* < 0.05). Leaf traits: ‘Area’, LDMC (‘Leaf dry matter content’), L/W (‘Length/Width ratio’), LMA (‘Leaf mass per area’) and ‘Perimeter’. Root traits: RDMC (‘Root dry matter content’), SRL (‘Specific root length’) and AvgDiam (‘Diameter’) for fine and thick roots, ‘Total lignin content’ and AIR (‘Acid insoluble residue’). Plant traits: PDMC (‘Plant dry matter content’), LMF (‘Leaf mass fraction’), SMF (‘Stem mass fraction’) and RMF (‘Root mass fraction’).

The Principal Component Analysis (PCA) carried out on five of the leaf traits considered (namely ‘Area’, ‘Perimeter’, LDMC, LMA and L/W) explained more than 80% of the total variance (PC1 = 59% and PC2 = 21.3%), with a major contribution of ‘Perimeter’ and ‘Area’ for the first PCA axis (27.4% and 21.6%, respectively), and LMA and ‘Area’ for the second axis (47.8% and 25.3%, respectively) (Figure 2A). The PCA performed with ‘tolerance’ as factor showed two separated groups, as confirmed by the Tukey test on the first PCA dimension (Figure 2A). The tolerant cultivars displayed higher leaf ‘Area’ and ‘Perimeter’ (PC1), and higher LMA (PC2), than the susceptible ones (Figure 2A).

The PCA performed with ‘variety’ as factor showed that ‘Frantoio’ and ‘Empeltre’ clustered together, confirming the ANOVA results (Figure S1A,B). This group was clearly separated from that formed by ‘Picual’, ‘Lechín de Sevilla’ and ‘Nevado fino’, that clustered together, and from ‘Hojiblanca’ which showed low differences in leaf traits.

### 2.3. Differences in Root Functional Traits

**Figure 3:**
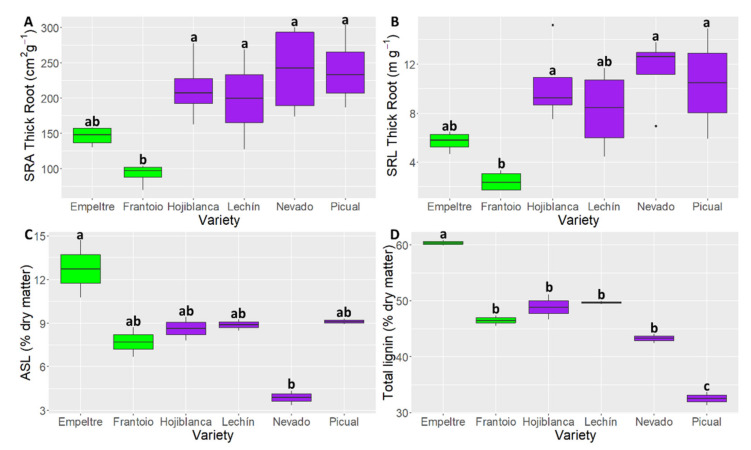
Boxplots showing median values of the ANOVA analysis of the most statistically significant root traits: SRA Thick Root (‘Thick root specific area’) (**A**), SRL Thick Root (‘Thick root specific length’) (**B**), ASL (‘Acid soluble lignin’) (**C**) and ‘Total lignin content’ (**D**). Tolerant cultivars are represented in green color while the susceptible ones are shown in purple color. Letters indicate Tukey HSD post hoc tests at the *p* < 0.05 level, following ANOVA.

The ANOVA analysis showed more significant differences among varieties when comparing thick rather than fine roots (Table 1). Indeed, ‘Frantoio’ exhibited significant difference with four of the analyzed cultivars (Hojiblanca, Lechín de Sevilla, Nevado fino and Picual) for ‘SRA Thick Root’ and with three of them (Hojiblanca, Nevado fino and Picual) for ‘SRL Thick Root’, both functional traits showing the lowest values in this tolerant cultivar (Figure 3A,B). Noteworthy, these thick root traits also exhibited significant differences for the ANOVA analysis performed between tolerant and susceptible cultivars (Table 1, Figure 3B), the tolerant varieties showing smaller values. With regard to the fine roots, the ANOVA analysis performed with ‘tolerance’ as factor showed that the tolerant varieties had larger (*p* < 0.001) values in fine root diameter compared with the susceptible ones, while the former cultivars showed larger (*p* < 0.05) ‘SRL fine root’ values (Table 1).

The ANOVA analysis of lignin content showed significant differences (*p* < 0.05) for all the indices analyzed when considering the factor ‘variety’. Instead, when taking into account the factor ‘tolerance’, only ASL and ‘Total lignin content’ were significantly different (*p* < 0.05) (Table 1). ‘Empeltre’ showed significantly higher values than ‘Nevado fino’ for ASL (Figure 3C), and with all varieties for the ‘Total lignin content’. For this latter trait, ‘Frantoio’ also showed significantly higher values than the susceptible variety ‘Picual’ (Figure 3D).

The root traits taken into account for the PCA explained 79.1% of the total variance (PC1 = 52.9% and PC2 = 26.2%), with a major contribution of SRL and SRA of both fine (19.7 and 19.4 %, respectively) and thick (14.6 and 17.1 %, respectively) roots for the first axis, and of ‘Total lignin content’ and SRA Fine Root for the second axis (28.0 and 11.7 %, respectively) (Figure 2B). The PCA analysis performed with ‘tolerance’ as factor showed two separated groups. Tolerant and susceptible cultivars showed the center of their ellipses on the two opposite sides of the PC1 axis, as confirmed by the Tukey test performed on the two PCA dimensions (Figure 2B). The tolerant cultivars showed lower SRL of fine and thick roots, lower SRA for thick roots, higher ‘Fine root diameter’, and higher ‘Total lignin content’ than susceptible plants (Figure 2B).

The PCA performed with ‘variety’ as factor (Figure S2A,B) did not show a clear difference for the evaluated traits, especially for the first axis (PC1) as showed by the Tukey test performed on the PCA dimensions, confirming the trends observed by ANOVA analysis (Figure 3).

### 2.4. Differences in Plant Functional Traits

**Figure 4:**
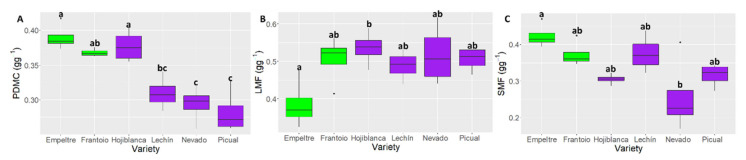
Boxplots showing median values of the ANOVA analysis of the most statistically significant plant traits: PDMC (‘Plant dry matter content’) (**A**), LMF (‘Leaf mass fraction’) (**B**) SMF (‘Stem mass fraction’) (**C**). Tolerant cultivars are represented in green color while the susceptible ones are shown in purple color. Letters indicate Tukey HSD post hoc tests at the *p* < 0.05 level, following ANOVA.

The ANOVA analysis of traits related to plant biomass showed significant differences among the olive varieties studied (Table 1). The PDMC showed significant differences (*p* < 0.001) between ‘Nevado fino’ and ‘Picual’, these varieties displaying the lowest values, and ‘Empeltre’, ‘Frantoio’ and ‘Hojiblanca’ that showed the highest values (Figure 4A). In contrast, and for the ‘Leaf mass fraction’, ‘Empeltre’ showed the lowest value (0.32 g∙g^−1^) while ‘Hojiblanca’ exhibited the highest one (0.58 g∙g^−1^), these cultivars being the only ones that displayed significant differences for this trait (*p* < 0.05) (Figure 4B). The ‘Stem mass fraction’ (SMF) showed a rather similar trend to that observed for PDMC, but significant differences (*p* < 0.05) were only observed between ‘Empeltre’ and ‛Nevado fino’, the latter cultivar showing the lowest value (0.17 g∙g^−1^) (Figure 4C). Interestingly, PDMC, LMF and SMF showed significant differences also in the ANOVA analysis performed with ‘tolerance’ as factor (Table 1), the tolerant varieties showing higher PDMC and SMF values (*p* < 0.01), but lower LMF (*p* < 0.05).

The plant traits taken into account for the PCA explained 71.7% of the total variance (PC1 = 39.2% and PC2 = 32.5%), with a major contribution of fine and thick root proportion for the first axis (30.5% and 28.3%, respectively) and of SMF and RMF for the second axis (43.8% and 19.6%, respectively) (Figure 2C). The PCA analysis performed with ‘tolerance’ as factor showed a separation between tolerant and susceptible groups on the first axis, confirmed by the post hoc test. The tolerant cultivars showed a higher PDMC and SMF than the susceptible ones (Figure 2C).

The PCA performed with ‘variety’ as factor showed that olive varieties clustered in two groups: ‘Picual’, ‘Lechín de Sevilla’, ‘Hojiblanca and ‘Nevado fino’ on one side and ‘Frantoio’ and ‘Empeltre’ on the other (Figure S3A,B).

### 2.5. Correlation among Functional Traits

**Figure 5:**
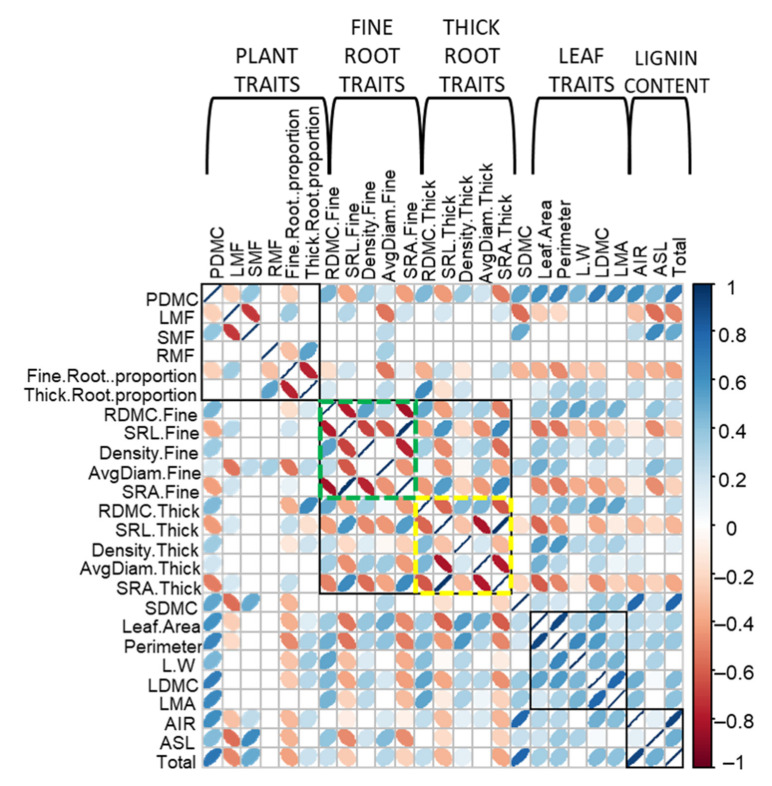
Bivariate correlation matrix between functional traits of the six olive varieties (Empeltre, Frantoio, Picual, Hojiblanca, Nevado fino y Lechín de Sevilla) here under study. Left and right ellipse inclination indicate significant negative and positive correlation, respectively. Blue and red colors indicate significant positive and negative correlations, respectively (*p* < 0.05). A high correlation coefficient is indicated with thin ellipses. Black squares group all functional traits of a specific plant organ (i.e., root, leaf and the entire plant) or trait (i.e., lignin content). Green and yellow squares group the functional traits for fine and thick roots, respectively.

The analyzed olive functional traits showed different relationships among them. Concerning the correlation among varieties, leaf features exhibited almost exclusively positive relations (r^2^ ≈ 1) unlike entire plant and root traits. All leaf traits (‘Area’, ‘Perimeter’, ‘L/W ratio’, LDMC and LMA) were significantly (*p* < 0.05) and positively (0.79 > r^2^ > 0.93) related among them (Figure 5).

Regarding plant traits, SMF showed negative correlation with LMF and did not have significant relation with the root fraction. Moreover, plants with higher LMF also showed higher fine root proportion, while plants with higher RMF also showed a higher thick root proportion. Fine and thick root proportions showed negative relation between them (Figure 5).

With regard to roots, fine and thick root traits followed the same pattern, showing positive correlation between RDMC, ‘Density’ and ‘Diameter’ and negative correlations between SRL and all the other root functional traits, with the exception of the positive relation between fine and tick SLR (Figure 5).

Finally, considering tolerant and susceptible cultivars as separate groups, some interesting differences were found for plant and root traits (Figure S4). Tolerant varieties showed negative correlation between PDMC and LMF, while the susceptible ones showed the opposite trend. The same was observed for the correlation between LMF and fine and thick root proportion, with tolerant cultivars showing positive correlation in the first case and negative in the second one. Meanwhile, susceptible varieties showed the opposite trend. For the root traits, tolerant cultivars showed positive correlation between RMF and lignin indices ASL and ‘Total lignin content’, while susceptible plants showed negative relations between these traits. Finally, both tolerant and susceptible cultivars showed positive correlation between SRL and SRA for both fine and thick roots (Figure S4).

## 3. Discussion

Understanding the differences in functional traits of olive cultivars differing in their tolerance/susceptibility to VWO would help to unravel whether (and to what extent) leaf, stem, root and/or whole plant characteristics are related to the ability to tolerate or succumb to *V. dahliae* infection. This knowledge could be included among the selection criteria in breeding programs for VWO resistance. Moreover, the information generated will be valuable for improving the efficiency of available disease management strategies and could even be used as disease predictive tool. Therefore, the aim of this study was to explore differences of selected functional traits among six olive varieties widely known for their different tolerance level to VWO.

The tolerant and susceptible cultivars here under study showed significant differences in several functional traits. Thus, the summary of the most remarkable differences found were: (i) ‘Area’, ‘Perimeter’ and LDMC for the leaves; (ii) SDMC for the stems; (iii) SRL of fine and thick roots, ‘Diameter’ of fine roots and SRA of thick roots, as for the belowground organ concerns; and (iv) PDM, SMF and LMF for the entire plant.

Leaf area plays an important role in photosynthesis, light interception, water and nutrient use, growth, and yield potential [41]. This functional trait, along with leaf perimeter (to a lesser extent) is the most important variable explaining the difference between tolerant and susceptible cultivars in the first axis of the PCA analysis. Moreover, ANOVA analysis enabled to distinguish between varieties with high (‘Frantoio’ and ‘Empeltre’) and low (‘Picual’, ‘Nevado fino’, ‘Hojiblanca’ and ‘Lechín de Sevilla’) light interception capacity. Remarkably, these two groups of cultivars also differed in VWO tolerance. The results observed for LMA supported this finding. Indeed, this functional trait showed a trend to higher values (albeit not statistically significant) for ‘Frantoio’ and ‘Empeltre’ plants (VWO tolerant) compared with the other cultivars except for ‘Hojiblanca’ (all qualified as VWO susceptible). LMA is a key functional trait and an important indicator of plant strategies [42]. The ratio between leaf dry mass and leaf area (i.e., LMA) can be understood as the leaf-level cost of light interception. The leaf dry mass contains a huge number of compounds (minerals, organic acids, total non-structural carbohydrates, total structural carbohydrates, soluble phenolics, proteins, lignin and lipids). This mass augments upon increasing LMA. This implies that high-LMA species have larger concentrations of these compounds than low-LMA species. Therefore, the latter species tend to have a fitness advantage under high-resource conditions and are typically found in productive habitats. In contrast, high-LMA species have a fitness advantage under adverse growing conditions and are typically found in unproductive habitats. Plants with high LMA not only have a greater lifespan of leaves but also of roots, thereby conserving acquired nutrients and carbon more efficiently [43].

Concerning entire plant traits, SMF and LMF explained a large percentage of the variance between tolerant and susceptible cultivars. This is a very interesting outcome because these functional traits provide information about plant strategy; that is, on how the plant allocates its resources. Stem biomass relative to total plant biomass reveals resource allocation for stem functions such as supporting leaves and flowers, transporting water and nutrients and light acquisition. Inversely, leaf biomass relative to total plant biomass reveals the investment in aboveground biomass, for the performance of photosynthesis and respiration processes [44]. VWO-susceptible plants presented a higher investment in leaf biomass, while tolerant cultivar showed larger biomass allocation in the stems (Figure 4B,C). RMF showed greater (albeit non-significant) values for susceptible cultivars than for tolerant plants (data not shown). It is tempting to speculate that larger biomass and resource allocation in the stem fraction of tolerant plants could be associated with the lower or null development of VWO symptoms in these cultivars when infected by *V. dahliae*. The different reaction to the pathogen’s invasion observed for tolerant and susceptible plants has been associated with the rate of distribution of *V. dahliae* within the plant. Besides, it has been demonstrated that in the absence of symptoms, or in plants showing mild VWO symptoms, the transversal growth of the pathogen is restricted in the xylem [45,46]. The mayor stem biomass allocation of tolerant cultivars could be related with the ability of these cultivars to slow down fungal dispersion, unlike susceptible cultivars, which invest more resources in the leaf biomass. Poorter and coworkers [38] concluded that the relative amount of biomass present in the various organs is not fixed but may vary over time, across environments and among species, and that our knowledge on the variation of biomass allocation is rather scant. A deeper understanding of biomass allocation patterns would be instrumental not only for plant ecology but also for elucidating possible links between this functional trait and increased tolerance to pathogens.

Concerning the root traits, significant differences between tolerant and susceptible cultivars were found for fine root diameter, SRA of thick roots, and SRL of fine and thick root (Table 1). The first trait is related to the root morphology, particularly with root penetration ability and hydraulic conductivity [39]. As demonstrated by Olmo and coworkers [47] fine roots showed the most dynamic diameter category since they can change faster upon environmental fluctuations. SRA is defined as the root surface area per root mass [48] and may be considered as one of the best general characteristics of root structure [49]. Its variation among species is usually similar to that of SRL [49,50], as we found among the analyzed olive varieties and, above all, between VWO-tolerant and VWO-susceptible cultivars (Table 1; Figure 3A,B, Figure S4). SRL indicates how much root length is built per unit of root mass [51,52]. Increasing the SRL enhances the root–soil interface and, hence, the root absorption potential [53] what should be an advantage when water or nutrients are limited [39]. Usually, roots with high SRL present small average diameter, as found in this work. Indeed, susceptible cultivars showed higher SRL and smaller root diameter, both for fine and thick roots, compared to tolerant plants. In general, thin roots have a high specific root length. Since water and nutrient uptake is based more upon root length than on its mass, one might conclude that species of high SRL invest their root biomass more efficiently than species of low SRL [54]. Eissenstat and Caldwell [55] showed that roots with high SRL (small diameter) are more plastic in lateral root proliferation. Moreover, several studies demonstrated that species showing high SRL produce ‘root length’ more rapidly and obtain greater root length densities than species with lower SRL [54,56,57]. In our study, susceptible cultivars showed higher root SRL values for both fine and thick roots. This would imply that susceptible plants invest biomass more efficiently in root growth compared with the tolerant ones, and that they are more plastic in lateral root proliferation. In contrast, tolerant plants have thicker roots and fewer developments in length and lateral accretion. These results are in accordance with recent findings on the differential basal gene expression patterns observed in roots of extremely susceptible (ES) and tolerant olive varieties. Indeed, Ramírez-Tejero and coworkers [58] showed that gene expression in roots of ES cultivars was more devoted to growth and development processes than that of tolerant varieties, which invested more in other functions such as defense against pathogens. Moreover, our results are in agreement with previous observations by Leyva Pérez and coworkers [46] who already underlined a dissimilar root system architecture between tolerant (‘Frantoio’) and susceptible (‘Picual’) plants, ‘Frantoio’ roots being less branched than ‘Picual’ roots regardless the age of the sampled plants. Similarly, Chatzistathis and coworkers [8] showed that ‘Koroneki’ olive plants, classified as tolerant to *V. dahliae* [59], showed less branched roots and with fewer root hairs development and density, compared with the susceptible cultivar Kothreiki.

All the susceptible cultivars analyzed in this study presented similar root functional traits that greatly differed in the tolerant plants. It can be argued that an olive root system presenting more lateral branches, more plasticity and thin roots is more prone to be infected by *V. dahliae*. A more branched root system exposes a larger contact surface to the soil, thereby increasing the likelihood to interact with propagules of the pathogen. Moreover, thin roots, predominant in susceptible cultivars, might not be able to counter the pathogen attack as efficiently as thicker roots, more frequently present in tolerant cultivars. Since roots are the first point of contact for soil borne pathogens, it is plausible to think that differences in root architecture and composition may greatly determine olive performance against colonization and invasion by *V. dahliae.*

Another clear difference between tolerant and susceptible cultivars at the root level related to the lignin content (Table 1; Figure 3C,D). Lignin is a major phenolic polymer present in the secondary cell wall of vascular plants, providing strength and resistance to the cell wall [60]. The importance of lignin in plant defense is linked to its role as mechanical barrier restricting/hindering the entrance of pathogens [61]. The deposition of lignin in the plant cell wall not only provides an effective obstacle to mechanical penetration by fungi, but also physically shields cell wall polysaccharides from degradation by fungal enzymes. It also restricts the diffusion of enzymes and toxins from the fungus to the host, and of water and nutrients from the host to the fungus [62]. Evidence on the important role of the lignification of olive cell walls to counter the attack by pathogens is available. For instance, Sabella and coworkers [33] detected a significant increase in total lignin content only in stems of tolerant olive varieties infected by *Xylella fastidiosa* compared with healthy plants and susceptible varieties. Gharbi and coworkers [12] showed that the soluble lignin content in stems and roots of the olive variety Sayali, tolerant to *V. dahliae*, was significantly higher than the susceptible one ‘Chemlali’ before the inoculation with the pathogen. Moreover, in the presence of the pathogen, the tolerant variety showed a significantly higher and faster increase in the lignin content in roots compared with the susceptible cultivar. We earlier identified a unigene potentially coding for a Dirigent-like protein involved in lignification that showed a significantly higher basal upregulation in ‘Frantoio’ (tolerant) than in ‘Picual’ (susceptible) [46]. Overexpression of the *DIRIGENT1* gene in cotton, leading to an enhanced lignification process and hampered invasion by *V. dahliae*, was previously reported as well [63]. Accordingly, our results showed a higher lignin content in roots of the tolerant varieties compared with the susceptible ones.

Another functional trait deserving attention is the ‘Dry matter content’. Most of the different plant parts analyzed (i.e., leaf, stem, whole plant) showed significant differences between tolerant and susceptible cultivars for this trait. This was particularly true for ‘Picual’ (very susceptible to VWO) and ‘Empeltre’ (tolerant to VWO), which exhibited the smallest and largest values, respectively, for this trait in leaf, stem and the entire plant (Figure 1C,D and Figure 4A). The dry matter content in leaf is positively related with leaf thickness, a characteristic that plays an important role in leaf and plant functioning and that is associated with species’ strategies of resource acquisition and use. Leaf thickness influences the amount of light absorbed by a leaf and the diffusion pathway of CO_2_ through its tissues [64]. Negative relationships between leaf thickness and photosynthetic and growth rates have been observed. Thicker leaves have often been linked to increased longevity and construction costs [65]. LDMC is definitely negatively related to nutrient content and positively associated with leaf toughness, reduced palatability and leaf decomposition rate, all characteristics linked with high content of lignin, fiber and silica [66]. Interestingly enough, the results for LDMC were very similar to those for LMA (‘Picual’ showed the lowest values while ‘Frantoio’ and ‘Empeltre’ the highest ones) (Figure 1B), which is another functional trait related to leaf thickness and light interception. In our work, VWO-tolerant varieties showed higher ‘Dry matter content’ for leaf (LDMC), stem (SDMC) and the whole plant (PDMC). We thus hypothesize that larger contents of dry matter could be related to better tolerance to diverse environmental stress [67], including higher tolerance to *V. dahliae* pathogen infection.

Relevant differences were found not only between tolerant and susceptible cultivars, but also among varieties. This was an interesting finding because the analysis of intraspecific functional traits has been often overlooked/neglected compared with studies carried out at the interspecific level [19]. Only recently the has intraspecific variability (i.e., phenotypic and genetic differences among individuals within a species) been gaining importance in understanding ecology and evolutionary biology [22,23,68]. Functional diversity responses to environmental fluctuations can result from changes in the species composition and/or intraspecific trait reaction to the environment [20]. De Bello and coworkers [20] explained that a decrease in plant functional diversity due to fertilization could be related to either a change in species composition alone or to just intraspecific variability. Nevertheless, functional diversity response very often depends on a combination of both. The high functional variability found in our study could be attributed to the long domestication and selection processes experienced by olive cultivars to increase their agronomical and commercial values. The selection of different characteristics (i.e., fruit size, oil yield, drought resistance) in different cropping areas has produced a large number of varieties over time, thereby generating a high degree of diversity [69]. Jiménez-Ruiz and coworkers [11] have recently unveiled the evolution of the olive genome during its domestication process. They found a neat geographical clustering of the olive cultivars under study and a clear separation between southern and northeastern Spanish olive varieties, which indicates a strong local selection for this species. Therefore, we encourage further studies regarding functional traits in olive varieties, not only to elucidate their links with disease resistance/susceptibility but also to understand their importance in intervarietal diversity and their relationship with environmental variables.

## 4. Materials and Methods

### 4.1. Olive Varieties and Plant Tissue Manipulation

Six olive varieties differing in VWO tolerance level were selected for this study (Table 2). All olive plants (18–24 months old), originated from stem cuttings of certificated mother plants purchased in a commercial nursery located in Córdoba province (southern Spain). The plants were grown in 1L PVC pots filled with a peat-based substrate containing Osmocote Exact standard 12–14M (1 g/L) (ICL Specialty Fertilizers-Iberia, Murcia, Spain), a universal slow-release fertilizer, and calcium carbonate (0.5 g/L), with a final pH of 7.5 ± 0.5. The plants were maintained in a greenhouse for three weeks under natural lighting, with a temperature of 23 ± 4 °C and a relative humidity ranging from 40% (day) to 80% (night). After this acclimation period in the greenhouse, four plants per variety were sampled. Each plant was carefully uprooted from the pot and the aboveground part and the root system were then separated (Figure S5). The aboveground part was divided into stems and leaves and stored in plastic bags (5 °C) for a few hours until use. Roots were gently washed from the soil under tap water, avoiding the loss of or damage to tissue, and then split into two portions for: (i) measurement of functional traits, and (ii) quantification of lignin content (see below). Special care was taken to sample representative portions of the entire root system (i.e., young and old roots). Lignin quantification was carried out collecting about 20 g of roots, which were rapidly frozen in liquid nitrogen and stored at −80 °C until further processing. The remaining roots were kept in a plastic bag with water and preserved at −20 °C until being used for functional traits analysis.

**Table 2:** Classification of the selected olive cultivars according to their susceptibility to Verticillium wilt of olive caused by the defoliating pathotype of *Verticillium dahliae*.

Cultivar	Origin	Susceptibility	Reference(s)
Empeltre	Spain	T	[3,70]
Frantoio	Italy	T	[13,46,71,72]
Hojiblanca	Spain	E	[9,70]
Lechín de Sevilla	Spain	S	[3]
Nevado fino	Spain	S	[3,73]
Picual	Spain	E	[13,46,71,74]

T = tolerant, S = susceptible, E = extremely susceptible.

### 4.2. Measurements of Functional Traits of Leaves, Stems and Roots

A subsample of leaves per plant was selected to measure leaf functional traits. This leaf subsample (approximately 20% of the leaf biomass) were scanned (ADF HP Scanjet 6300c; Hewlett-Packard, CO, USA) and leaf area, perimeter, green density, length and width were calculated using Image Pro 4.5 (Media Cybernetics Inc. Rockville, MD 20852 USA). Finally, the fresh and dry (70 °C, 48 h) mass of all the leaf fractions was measured. The cuttings and the stems were separated, and their fresh and dry (70 °C, 48 h) masses were determined. A fraction of the root biomass of each plant stored at −20 °C was taken to analyze the functional traits. This fraction represented about 20 ± 5% of total root biomass (mean ± SD), and, in morphological terms, represents one of the main roots linked to the root-stem connection. The roots were classified in two different diameter categories: thick roots (diameter > 2 mm) and fine roots (<2 mm) and their fresh masses was determined. Root segments were placed on a scanner (Epson Expression 164, Seiko Epson Corp., Nagano-Ken, Japan) in a transparent plastic tray filled with water and analyzed using WinRHIZO Pro v.3.10b (Regent Instrument Inc., Quebec, Canada). The output of the software gives the following measurements: mean root diameter (mm), total root length (cm), surface area (cm^2^), root volume (cm^3^) and length of each diameter class (between 0 and 4.5 mm). Based on these measurements different functional traits were calculated (Table 1).

### 4.3. Determination of Acid-Insoluble Additionally, Acid-Soluble Lignin Content

The acid-insoluble residue (Klason lignin) was determined gravimetrically [75], and the acid-soluble lignin was determined spectrophotometrically [76,77]. The root tissues stored at −80 °C were ground in liquid nitrogen and dried at 105 °C until a constant mass was achieved. To determine the lignin content, 0.3 g of the dry material was digested (72% H_2_SO_4_, 60 °C, 30 min) under occasional stirring. After complete digestion, the reaction mixture was diluted (4% H_2_SO_4_) and autoclaved at 121 °C for 1 h, followed by filtration through a glass fiber filter to separate the soluble and insoluble fractions. The acid-soluble lignin (ASL) was determined in the filtrate by spectrophotometry at 205 nm to avoid acid degradation products [78]. The remaining solid residue was dried overnight at 105 °C and the acid-insoluble residue (AIR) was calculated by the difference in the mass of the sample after and before the acid hydrolysis.

### 4.4. Statistical Analysis

To assess whether differences existed in the traits measured for (a) the six different olive varieties, and (b) the different level of susceptibility to VWO of the cultivars, data were analyzed with one-way ANOVA (R function aov), considering separately these two factors: ‘variety’ and ‘tolerance’. We considered only two levels for the ‘tolerance’ factor: ‘tolerant’, which included the tolerant (T) cultivars Frantoio and Empeltre, and ‘susceptible’, which grouped susceptible (S) and extremely susceptible (E) cultivars Picual, Hojiblanca, Lechín de Sevilla and Nevado fino (Table 2). To analyze differences among varieties or between the tolerant/susceptible groups, a Tukey post hoc test was used with a *p* level of 0.05 (R package agricolae) [79]. Much as for the ANOVA analysis, Principal Component Analysis (PCA) was carried out to evaluate how leaf, root, stem and entire plant traits differed considering the two different category variables (factor): ‘variety’ and ‘tolerance’ (R package factoextra) [80]. To study the relationships among traits, a correlation matrix of all traits taken into account in the PCA analysis was graphically represented in two different ways. Firstly, analyzing the existing correlations among varieties, and secondly, examining the correlation of tolerant and susceptible cultivars separately. To generate the correlation matrixes, and the corresponding figures, the function corrplot of the Corrplot package of the R software was used [81]. All the statistical analyses were performed using the statistical software R [82].

## 5. Conclusions

This study suggests that the tolerance of olive varieties to *V. dahliae* is influenced by specific functional traits of leaves, shoots and, especially, roots. Moreover, it confirms the important role of the root lignin against pathogen attack. For several plant species the genetic and hormonal control of root anatomy has already been described under different environmental and stress conditions. However, information regarding olive root system and its relationship with tolerance/susceptibility to *V. dahliae* is almost null. Thus, a more in-depth study will be needed to elucidate the relation between olive root architecture and *V. dahliae* resistance, from both genetic and functional points of view, and considering presence and absence of the pathogen. Studies including more varieties are encouraged to comprehensively understand the mechanisms of resistance to VWO. Furthermore, the knowledge acquired by these studies would be of relevance in breeding for resistance and disease management strategies.

## Figures and Tables

**Table 1:** Results of ANOVA analyses for different plant traits using ‘variety’ or ‘tolerance’ as factors.

Plant Part	Trait	Abbreviation	Units	Explained Variance (‘variety’) (%)	Explained Variance (‘tolerance’) (%)
Leaf	Area	Area	cm^2^	62.97 *** ^1^	97.11 ***(+) ^1^
	Perimeter	Perimeter	cm	50.43 **	92.14 **(+)
	Green density	Green density	-	72.40 **	2.83
	Length/width ratio	L/W	-	73.05 **	8.12
	Leaf dry matter content	LDMC	g∙g^−1^	69.34 **	87.78 *(+)
	Leaf mass per area	LMA	g∙m^−2^	74.05 ***	9.38
Stem	Stem dry matter content	SDMC	g∙g^−1^	68.13 ***	85.18 *(+)
Plant	Plant dry matter content	PDMC	g∙g^−1^	89.43 ***	92.65 **(+)
	Leaf mass fraction	LMF	g∙g^−1^	62.80 *	57.84 *(-)
	Stem mass fraction	SMF	g∙g^−1^	72.27 *	87.63 **(+)
	Root mass fraction	RMF	g∙g^−1^	49.52	11.07
	Fine root proportion	Fine root prop.	g∙g^−1^	58.73	24.31
	Thick root proportion	Thick root prop.	g∙g^−1^	24.78	0.07
Fine root	Fine root specific length	SRL Fine Root	m∙g^−1^	47.56	67.55 *(-)
	Fine root tissue density	Density Fine Root	g∙cm^−3^	44.46	62.85
	Fine root average diameter	AvgDiam Fine Root	cm	67.60 ***	78.91 **(+)
	Fine root specific area	SRA Fine Root	cm^2^∙g^−1^	60.32	12.97
	Fine root dry matter content	RDMC Fine Root	g∙g^−1^	62.28	43.51
Thick root	Thick root specific length	SRL Thick Root	m∙g^−1^	45.16 **	85.58 ***(-)
	Thick root tissue density	Density Thick Root	g∙cm^−3^	17.91	19.37
	Thick root average diameter	AvgDiam Thick Root	cm	42.45	51.94
	Thick root specific area	SRA Thick Root	cm^2^∙g^−1^	51.19 **	90.56 ***(-)
	Thick root dry matter content	RDMC Thick Root	g∙g^−1^	1.42	28.63
Root lignin content	Acid insoluble residue	AIR	% dry matter	97.04 ***	68.76
	Acid soluble lignin	ASL	% dry matter	86.17 *	85.72 * (+)
	Total lignin	Total lignin	% dry matter	97.19 ***	84.6 * (+)

^1^ The explained variance percentage by olive variety (fifth column) or by tolerance (sixth column) factor (100 × SSfactor/SS total) and the level of significance (*, *p* < 0.05; **, *p* < 0.01; ***, *p* < 0.001) are shown (significant values are in bold). For tolerance, (+) means higher value for tolerant and (-) means lower value for this trait. SS represents the sum of squared differences from the mean.

## Data Availability

All data required to reproduce the results presented in this study can be found in the article.

## Supplementary Materials

The following information is available online at https://www.mdpi.com/article/10.3390/plants10061079/s1, Figure S1: Principal Component Analysis (PCA) of leaf traits (A) performed with ‘variety’ as factor and (B) the contribution of variables on the main two axes of PCA. Different letters indicate significant differences between groups (Tukey test, *p* < 0.05). The colored lines indicate the mean of the scores of the groups along dimension 1 and 2 of the PCA, when significant differences exist (LMM, *p* < 0.05). Leaf traits: Area, LDMC (‘Leaf dry matter content’), L/W (‘Length/Width ratio’), ‘Perimeter’ and LMA (‘Leaf mass per area’); Figure S2: Principal Component Analysis (PCA) of root traits (A) performed with ‘variety’ as factor and (B) the contribution of variables on the main two axes of PCA. Different letters indicate significant differences between groups (Tukey test, *p* < 0.05). The colored lines indicate the mean of the scores of the groups along dimension 1 and 2 of the PCA, when significant differences exist (LMM, *p* < 0.05). Root traits: RDMC (‘Root dry matter content’) of fine roots, SRL (‘Specific root length’) of fine and thick roots, SRA (‘Specific root area’) of fine and thick roots, ‘Total lignin content’ and AIR (‘Acid insoluble residue’); Figure S3: Principal Component Analysis (PCA) of plant traits (A) performed with ‘variety’ as factor and (B) the contribution of variables on the main two axes of PCA. Different letters indicate significant differences between groups (Tukey test, *p* < 0.05). The colored lines indicate the mean of the scores of the groups along dimension 1 and 2 of the PCA, when significant differences exist (LMM, *p* < 0.05). Plant traits: PDMC (‘Plant dry matter content’), LMF (‘Leaf mass fraction’), SMF (‘Stem mass fraction’), RMF (‘Root mass fraction’), fine and thick root proportion; Figure S4: Bivariate correlation matrix between functional traits of susceptible (lower-left triangle) and tolerant (upper-right triangle) olive cultivars. The circle dimension represents the correlation significance. Blue and red color indicate positive and negative significant correlations, respectively (*p* < 0.05); Figure S5. Scheme showing the olive tissues sampling strategy followed in this study. Each plant was carefully uprooted from the pot and aboveground organs and root systems were then separated. Subsequently, the aboveground part was divided into leaves and stems, and the latter was further separated into stems and cuttings. The root system was divided into fine and thick roots according to the diameter (see main text for details).
