# Supplementary figures and images for: Functional Traits of Olive Varieties and Their Relationship with the Tolerance Level towards Verticillium Wilt
Source: Plants (Basel). 2021 May 27;10(6):1079. doi: 10.3390/plants10061079 (PMC8230176; doi:10.3390/plants10061079)

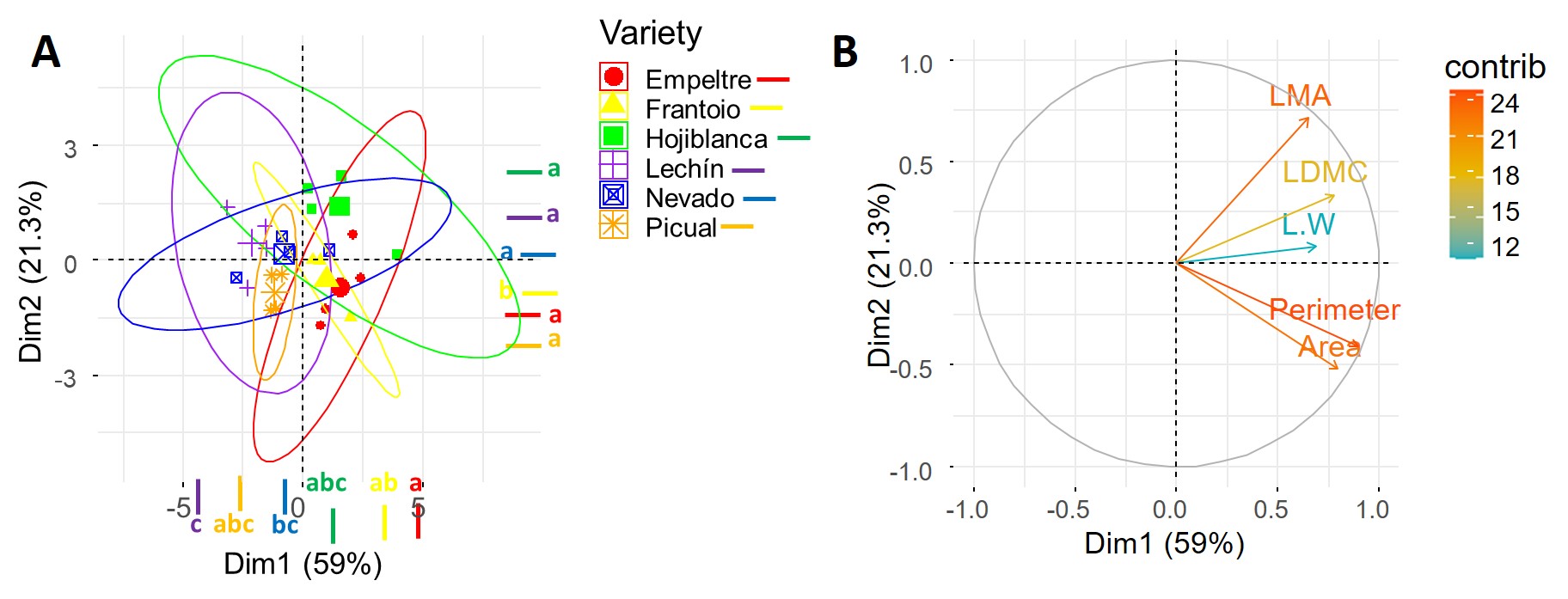

Supplement: Supplementary file 1 [file plants-10-01079-s001.zip › Figure S1.jpg]

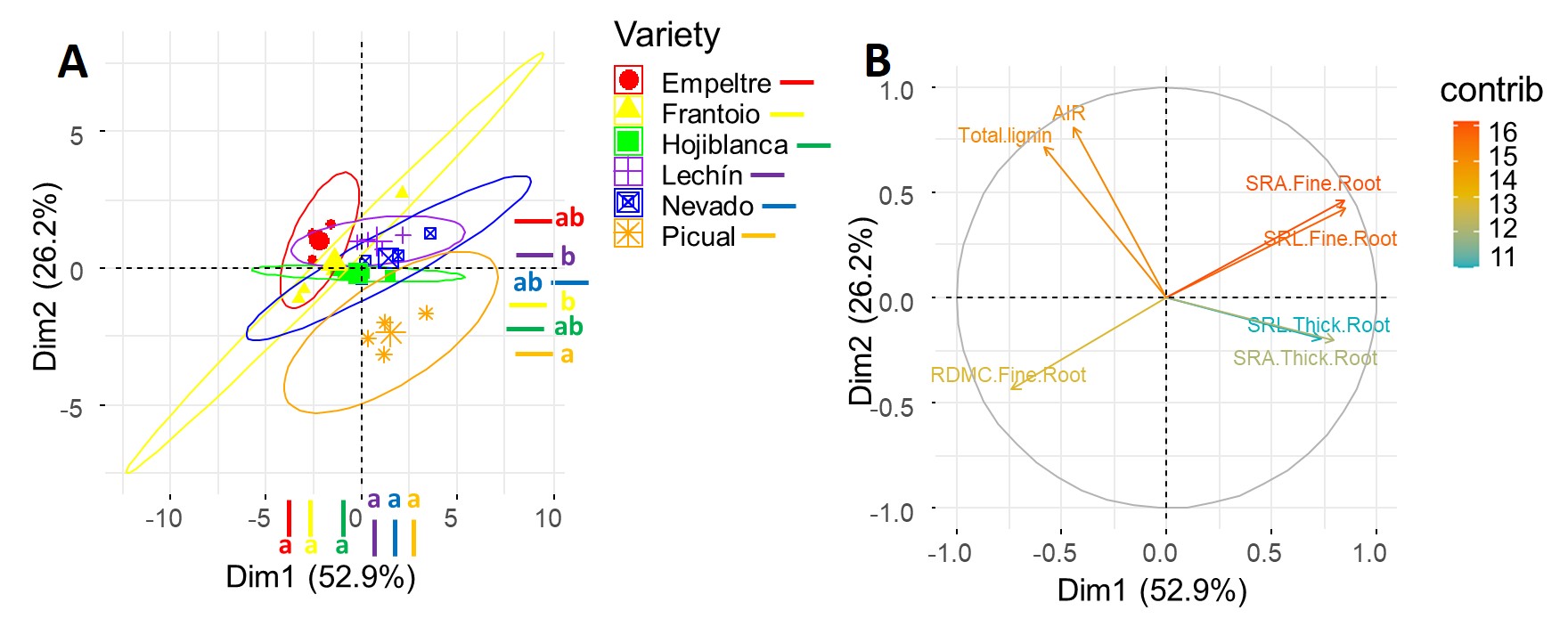

Supplement: Supplementary file 1 [file plants-10-01079-s001.zip › Figure S2.jpg]

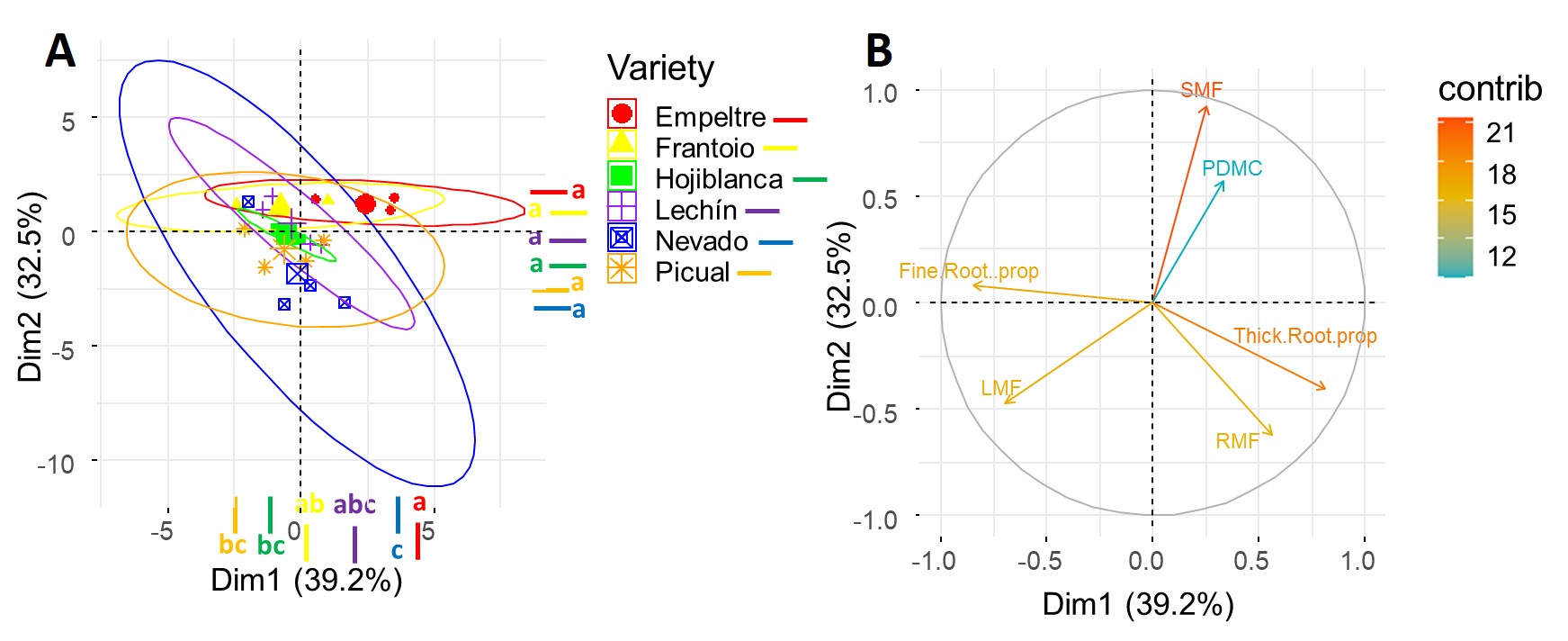

Supplement: Supplementary file 1 [file plants-10-01079-s001.zip › Figure S3.jpg]

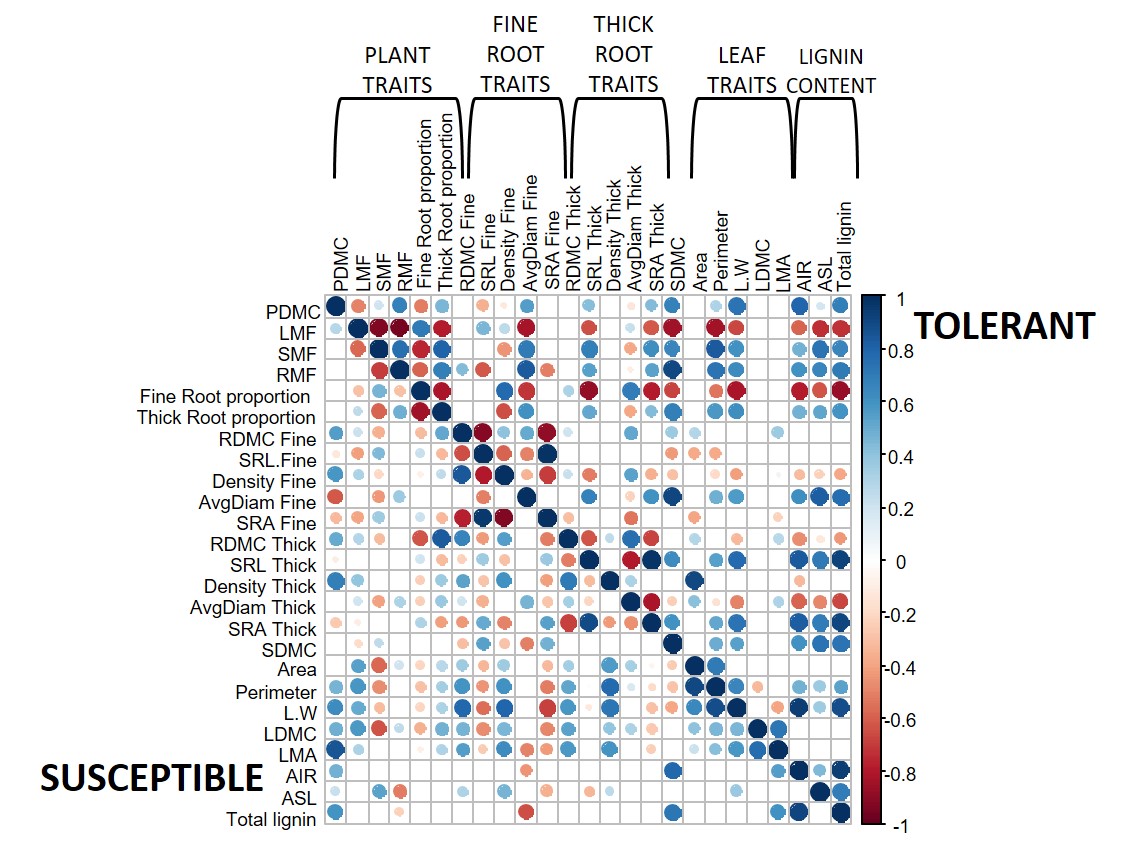

Supplement: Supplementary file 1 [file plants-10-01079-s001.zip › Figure S4.jpg]

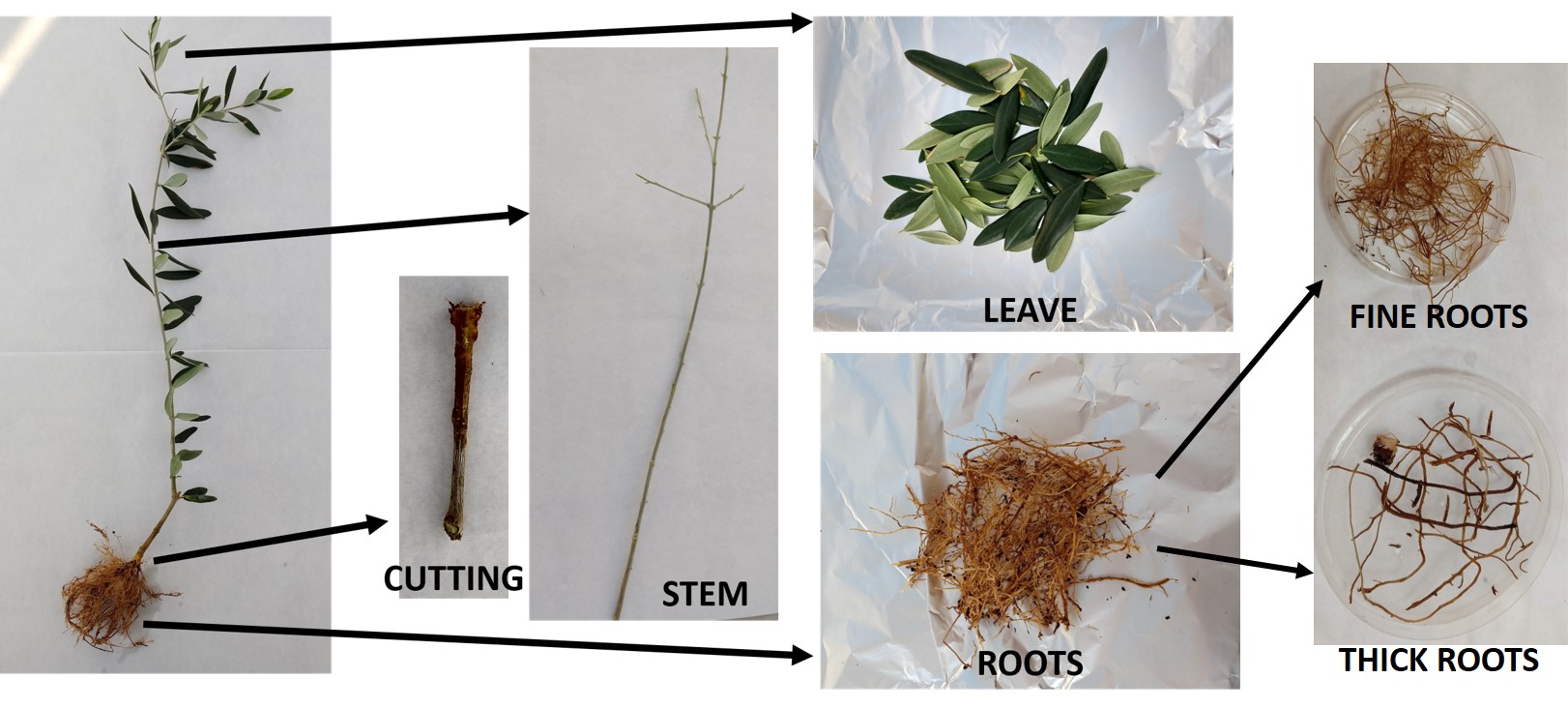

Supplement: Supplementary file 1 [file plants-10-01079-s001.zip › Figure S5.jpg]
